# Determining Biogenic Content of Biogas by Measuring Stable Isotopologues ^12^CH_4_, ^13^CH_4_, and CH_3_D with a Mid-Infrared Direct Absorption Laser Spectrometer

**DOI:** 10.3390/s18020496

**Published:** 2018-02-07

**Authors:** Teemu Kääriäinen, Craig A. Richmond, Albert Manninen

**Affiliations:** VTT Technical Research Centre of Finland Ltd., Vuorimiehentie 3, 02150 Espoo, Finland; craig.richmond@vtt.fi (C.A.R.); albert.manninen@vtt.fi (A.M.)

**Keywords:** stable isotopologues, biogas, direct absorption, biogenic determination, bioprocess monitoring, laser-based sensors

## Abstract

A tunable laser absorption spectrometer (TLAS) was developed for the simultaneous measurement of *δ*^13^C and *δ*D values of methane (CH_4_). A mid-infrared interband cascade laser (ICL) emitting around 3.27 µm was used to measure the absorption of the three most abundant isotopologues in CH_4_ with a single, mode-hop free current sweep. The instrument was validated against methane samples of fossil and biogenic origin with known isotopic composition. Three blended mixtures with varied biogenic content were prepared volumetrically, and their *δ*^13^C and *δ*D values were determined. Analysis demonstrated that, provided the isotopic composition of the source materials was known, the *δ*^13^C and *δ*D values alone were sufficient to determine the biogenic content of the blended samples to within 1.5%.

## 1. Introduction

As the utilisation of renewable energy sources increases globally, the safe and efficient integration of biofuels into existing energy infrastructures becomes particularly pertinent. Biogas is one such renewable energy source, with the potential to replace fossil fuels for applications such as power production and transportation fuel. Within Europe, this transition is supported by the Renewable Energy Directive 2009/28/EC [1], which promotes the use of renewable sources and thus drives the diversification of the natural gas supply. Due to increasing European biogas production [2,3], and to support the use of green gas, the European Committee for Standardization (CEN) has worked to establish technical specifications for both biogas (a mixture of methane and other gases, such as CO_2_) and biomethane (purified biogas with higher methane content), with the aim of allowing safe injection to the natural gas distribution grids, and for use as a fuel for vehicle engines [4].

The need to validate the biogenic content of gas samples consisting of blended biomethane and natural gas is included in such regulatory framework. One method for making this determination is the measurement of the radioactive carbon isotope, ^14^C. As fossil samples are free of ^14^C, the measured ^14^C content of a blended bio-fossil sample confirms the level of dilution of the biogenic methane. The biogenic fractions of CO_2_ emissions have been studied this way [5,6], successfully demonstrating the suitability of the ^14^C technique. Building on these studies, the first demonstration of ^14^C for determining the biogenic fraction of blended biogas and natural gas mixtures was reported by Palstra and Meijer [7]. For this study, the methane was converted to CO_2_, and the radiocarbon content determined by accelerator mass spectrometry (AMS). Very good results were achieved, with the uncertainty in the calculated biogenic fractions varying between ±0.7% and ±4.5%, depending on the sample. However, the instrumentation requirements and sample preparation time associated with AMS prevents online and real-time in situ analysis of methane samples.

Many of the practical limitations of ^14^C AMS measurements can be overcome by employing optical detection methods. Indeed, in recent years, a number of groups have published spectroscopic approaches for radiocarbon measurement. Detection limits approaching [8] and exceeding [9,10] ambient ^14^C levels have been reported. However, at present, these techniques are restricted to laboratory use only. In addition, they would require the conversion of methane to CO_2_, complicating the experimental procedure.

An alternate tool for determining the biogenic content is stable isotope analysis. The isotopic composition of natural gas and biogas is known to vary significantly [11,12,13,14], with the isotopic ratios providing a valuable tool for source discrimination. Importantly, experimental instrumentation can be simplified when employing optical detection methods to investigate the more abundant stable isotopologues of methane.

It is common for stable isotope measurements to be expressed in parts per thousand (‰) using ‘delta’ notation:

$$\delta =\left(\frac{R_{sample}}{R_{standard}}-1\right)\cdot 1000‰$$

where, in the case of methane, *R_sample_* and *R_standard_* are the ^13^C/^12^C or D/H (^2^H/^1^H) ratios of the sample and a standard, respectively. Generally, the Vienna Pee Dee Belemnite (VPDB) standard is used for ^13^C/^12^C ratios, whereas D/H ratios are referenced to the Vienna Standard Mean Ocean Water (VSMOW) standard.

To date, no studies have reported on the use of stable isotopes to determine the biogenic fraction in biogas and natural gas mixtures. Optical studies on stable methane isotopologues have commonly focussed on atmospheric applications with methane concentrations at ambient levels. Mid-IR absorption experiments have previously been used to report *δ*^13^C values [15,16,17,18], whilst recent studies ultilising quantum cascade lasers (QCL) around 7.7 µm have employed long optical path lengths [19] or preconcentration [20] to achieve sufficient sensitivity for simultaneous *δ*^13^C and *δ*D measurements. This has led to the availability of commercial instruments with excellent suitability to environmental monitoring applications, where methane levels range from atmospheric to slightly elevated concentrations. However, for applications where methane concentrations are significantly above ambient levels, such as biogas-related applications, there is clear potential for significant simplification of the experimental design, allowing for the development of more cost efficient instrumentation with a smaller footprint to better meet industrial needs.

The relevance of stable isotope measurements for biogas-related applications extends beyond biogenic determination. Lv et al. [21] utilised changes in methane *δ*^13^C values to characterise the dominant methanogenic pathways in biogas reactors, whilst further studies reported on how changes in the stable isotope composition of biogas during production can forecast reactor process instability [22] and failure [23]. As such, fast and compact instrumentation could greatly improve monitoring approaches within the biogas industry. In addition, there are potential applications for stable isotopologues measurements in examining reaction pathways in alternate CO_2_-neutral methane production methods such as artificial photosynthesis [24,25].

In this paper, we describe a compact and robust mid-infrared spectrometer developed to measure the *δ*^13^C and *δ*D values of methane both on-line and in real-time. The spectrometer is validated against methane samples of known isotopic composition, and the instrument performance is reported. Finally, we demonstrate the feasibility of determining the biogenic content of blended fossil and biogenic methane through stable isotope analysis.

## 2. Materials and Methods

**Figure 1:**
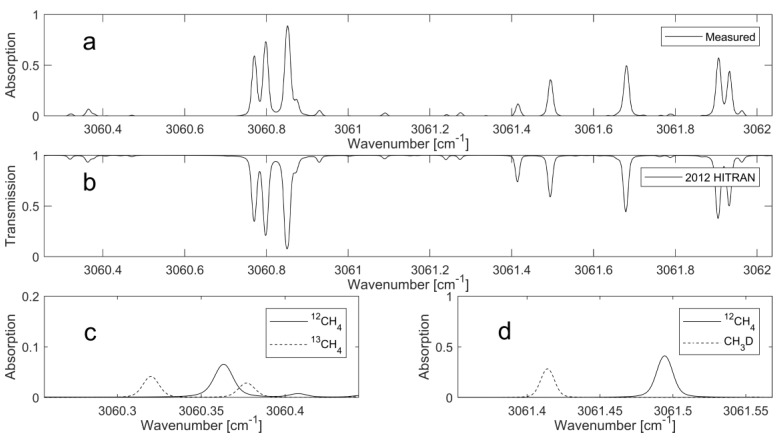
Absorption spectrum, averaged from 10 interband cascade lasers (ICL) current sweeps (**a**); simulated spectrum using the 2012 HITRAN database (**b**); simulated spectrum of the ^12^CH_4_ and ^13^CH_4_ line pair (**c**); and ^12^CH_4_ and CH_3_D line pair (**d**).

CH_4_ exhibits absorption features throughout the near and mid-infrared, with the strongest absorption band (ν3) at around 3 µm. The recent commercial availability of interband cascade lasers (ICL) emitting around the 3-micron region provides a compact light source for probing the strongly absorbing ro-vibrational transitions of CH_4_. Simulations based on the HITRAN 2012 database [26] reveal several possible spectral regions with access to the three most abundant CH_4_ isotopologues: main, ^13^C, and ^2^H (D), having natural abundances of 98.8%, 1.1% and 0.6‰, respectively. However, most of the regions with potential line combinations suffer from spectral overlap, significant variation in absorption intensities of the transitions of interest, or transitions that are too weak for practical measurement. In this work, two line pairs located at 3.27 µm were selected: a main and D line pair, neighboured by a main and ^13^C line pair. The combination of the two line pairs can be covered within one current sweep of the ICL. No interfering absorption from other hydrocarbons, such as ethane, was found in the HITRAN simulations. The simulated and measured spectra are shown in Figure 1.

The simulation is based on line parameters provided by the HITRAN 2012 database using a temperature of 305 K, pressure of 30 mbar, and 1-m pathlength. The line intensities of each isotopologue are based on their natural abundance. The measured spectrum (Figure 1a) was obtained from a methane sample (Gasum Oy, Kouvola, Finland, >97.5%) that was sourced from a biomethane production facility within Finland. Whilst the measured and simulated spectra in Figure 1 are in good agreement, there are noticeable discrepancies in the intensities for three lines at 3061.43 cm^−1^, 3061.24 cm^−1^, and 3061.32 cm^−1^. The first two correspond to CH_3_D transitions, and the final corresponds to ^13^CH_4_ transitions. Although uncertainties in the line intensities in the HITRAN database can account for some of the disagreements between the measured and simulated spectra, it is important to note that the methane sourced from biogenic origins is commonly depleted, relative to natural abundances, in heavier isotopologues [14]. This is the primary reason for the absorption intensity variations at the above-mentioned wavelengths.

For the transitions corresponding to the selected line pairs, the difference in the lower-state energies are 1344.7 cm^−1^ and 1534.4 cm^−1^ for D/H and ^13^C/^12^C, respectively [26]. The temperature dependency of the measured line pairs resulting from the difference in the ground state energies of the probed lines can be estimated [15] to be 25 ‰/K and 22 ‰/K for *δ*^13^C and *δ*D, respectively. Due to this dependency, the multipass cell was thermally insulated and stabilised with heating pads to limit the variations in temperature to less than 10 mK.

**Figure 2:**
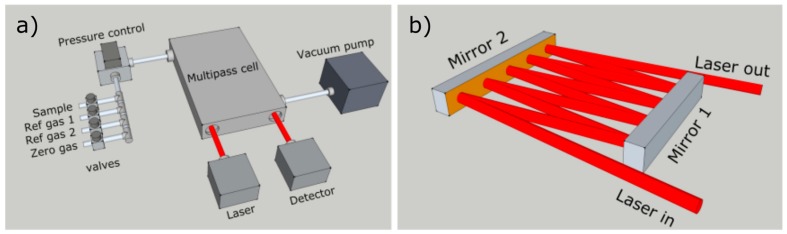
Schematic of the CH_4_ isotope analyser and gas handling (**a**); and the optical multipass cell (**b**).

The optical design, shown in Figure 2, was realised with simplicity and robustness as key factors. Two flat, rectangular, gold-coated mirrors, aligned parallel to each other, were enclosed within a multipass cell of 100 mm length, 50 mm width and 7 mm height, resulting in an overall sample volume of less than 50 mL (including tubing to connect the cell to the sampling line). The cell design allows for a simple layout and trivial optical alignment. With a collimated laser beam, no additional beam shaping optics are required, and the pathlength can be easily chosen between 0.4 and 3 m without the need of a visible trace laser. The optical pathlength was determined by observing the absorption depth of a known sample. With this design, up to 3 m of pathlength was tested without significant optical etalon fringes or stray light scattering. For the selected methane transitions in this work, 1 m of optical pathlength was utilised. This provided the best tradeoff between the different transition intensities, allowing for sufficient absorption depth of the weaker ^13^C line, whilst limiting the stronger absorbing D line (which is susceptible to nonlinearity issues at longer pathlengths).

Low sample volume was a prerequisite for the instrument design, in order to allow investigations on a miniaturised laboratory scale. To minimise sample consumption, the sample gas was enclosed in the optical multipass cell by solenoid valves. The schematic of the gas sampling system is shown in Figure 2. Prior to introducing a new sample, the sample line and the optical cell were purged with zero gas. In this work, the zero gas was ambient air, having generally less than 2 ppm of CH_4_. Relative to the methane concentration of the sample, and with respect to the optical pathlength, this level of methane is well below the noise level of the instrument. After purging, the sample cell and the sample line were evacuated to a pressure below 2 mbar. Performing this procedure twice proved sufficient to avoid sample contamination and memory effects. The sample, or one of the two reference gases, was introduced into the sampling line after the zero measurements. The pressure controller (MKS 640B) was used to regulate the pressure to 60 mbar, which was the optimal pressure for sufficient line separation whilst allowing adequate absorption. After a few seconds of stabilisation, the measurement was initiated. This sampling procedure resulted in a total of 60 s gas exchange time. Accounting for the experimental pressures, the equivalent volume of sample gas under ambient pressure was less than 3 mL.

Rapid laser sweep and fast data acquisition are essential for high-precision isotopic analysis using direct absorption spectroscopy. The fast laser sweeps transfer the detection bandwidth away from the dominating low-frequency components of 1/f noise, whilst fast data-acquisition is required to obtain a sufficient number of data points for proper spectral fitting (see McManus et al. [27] and references within). However, due to the use of high-concentration and undiluted sample gases, extreme precision was not as important as field capability. Sufficient performance (<1‰ precision) for the applications at hand was achieved with the selected USB DAQ card (NI USB-6356) providing 1.25 MS/s sampling rate.

A single-mode ICL (nanoplus Nanosystems and Technologies) emitting at 3.27 µm was employed as the light source. Current and thermal control was provided by a single module (Wavelength Electronics, LDTC2/2), powered by a linear power supply. The laser was scanned ~1.8 cm^−1^ by applying a current ramp at a 600-Hz repetition rate. An attempt to optimise the usable data points was made by sweeping the unused spectral region between the two line pairs more rapidly. In practice, this technique resulted in a trade-off between optimising the time spent on measuring the desired spectral features, and additional frequency chirp and temperature fluctuations due to the rapid current tuning rate changes. Optimal measurement stability was achieved with a linear ramp sweep over the whole spectral region covering both line pairs. A HgCdTe detector (Vigo System S.A, Warsaw, Poland, PVI-3TE-5) was placed after the measurement cell.

## 3. Results

**Figure 3:**
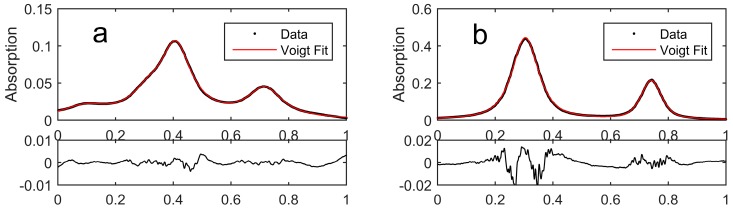
Measured spectrum (top) along a normalized scale with Voigt fit (red), and (below) residual (black) for line pairs ^13^C/^12^C (**a**) and D/H (**b**). The residual is calculated by subtracting the fitted values from measurement data.

The measured spectra are shown in Figure 3. The transitions were fit with a Voigt approximation [28] and second order polynomial to account for the background. For the D/H line pair, no interfering lines overlapped with the measured transitions. However, in order to determine the ^13^C/^12^C ratio, two transitions for each isotopologues needed to be fitted. The positions of weaker lines were fixed relative to the strongest ^12^CH_4_ transition. The residual shows the well-known Voigt w-shape that originates from non-ideal spectral shape representation. However, the magnitude of the residual is indicative of additional contributions from the instrumentation, most likely originating from insufficient detector bandwidth.

**Figure 4:**
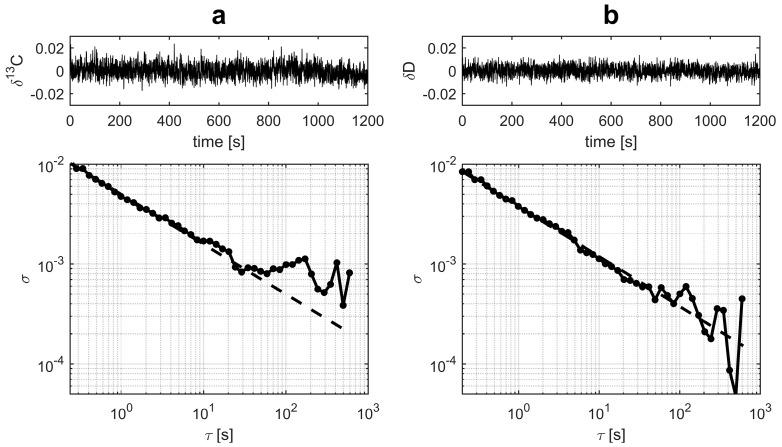
Typical Allan deviation plot for *δ*^13^C (**a**) and *δ*D (**b**) in CH_4_. The filled circles show the experimental data and the dashed line shows the theoretical 1/f random noise behavior.

Allan deviation analysis has become a standard benchmarking tool for laser instrument stability. Multiple time series were measured for the gas samples to study the stability of the instrument. A typical result is shown in Figure 4. The 1 s Allan deviations were less than 5‰ for *δ*^13^C and less than 4‰ for *δ*D. With 30 s averaging, the Allan deviations of both isotopic ratios were below 1‰. If referenced with two standards, a single analysis time was 240 s.

In order to determine whether gas exchange affected the stability, the same gas was introduced to the gas cell repeatedly. For this purpose, 20 s averages were plotted to observe the deviation (data not shown). An increase factor of two to three was induced in the standard deviation compared to the case where the static gas was measured without exchange. This decrease in performance was most likely due to the optical background change between the samples, which was caused by the pressure variations during gas exchange.

Two-point calibration was used to set the measured isotopic ratio values to the absolute *δ*-scale. The calibration gas was introduced between each measurement to correct for the instrumental drift. Two samples having different isotopic composition—one natural gas and one biomethane—were acquired and analysed with isotope ratio mass-spectrometry (IRMS) in the Stable Isotope Laboratory at the Max-Planck Institute for Biogeochemistry, according to methods previously described [29,30]. The isotopic composition of the biomethane sample was *δ*^13^C = −57.66 ± 0.06‰ VPDB, and *δ*D = −367.3 ± 2.6‰ VSMOW. The values for the fossil sample were −45.55 ± 0.06‰ and −203.3 ± 1.2‰, respectively.

To validate the final performance of the laser spectrometer, three mixtures with varying mixing ratios of the IRMS-analysed natural gas and biomethane were prepared volumetrically using calibration gas mixture standards [31], which were modified to use a stainless steel vessel at 10 bar as opposed to glass vessels. The resulting mixtures possessed biofractions of 73.4%, 50.3%, and 26.4%. The uncertainty of the volumetric dilution was less than 0.1% for all three mixtures.

**Figure 5:**
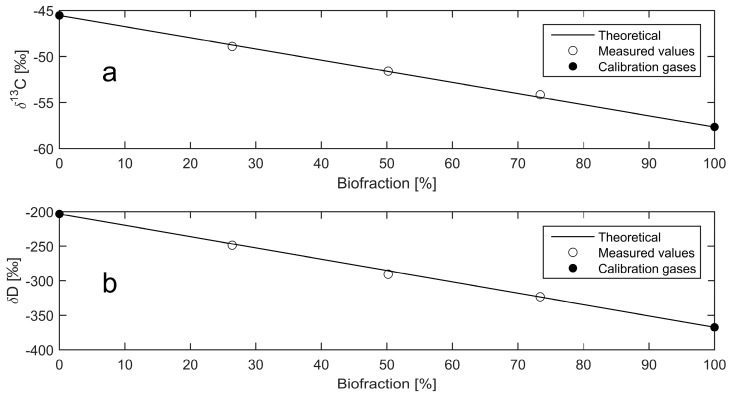
Biofraction determination with the developed laser spectrometer. The average of 100 consecutive measurements of *δ*^13^C (**a**), and 10 consecutive measurements of *δ*D (**b**), in CH_4_.

A custom-developed LabVIEW program was prepared to automatically switch between the sample and reference gases to achieve repeatable and consistent measurements for each sample. For each of the methane mixtures, the standard deviation for *δ*D using 10 sample analyses was below 2.6‰. For *δ*^13^C, the range of delta values between the bio and fossil samples was significantly smaller than for *δ*D. Thus, the standard deviation of the *δ*^13^C values must be significantly smaller than the *δ*D values in order to achieve the same sensitivity with regards to biofraction determination. As a result, 100 sample analyses were recorded for *δ*^13^C, achieving a standard deviation below 0.3‰ for each methane mixture. Figure 5 shows the measurement results obtained with the laser spectrometer. The solid line represents the expected isotopic ratio vs. CH_4_ biofraction. The biofraction calculated from the measured isotopic ratios agreed with the volumetric dilution to within 1.5% for both *δ*^13^C and *δ*D. This is comparable to the uncertainty range of 0.7–4.5% reported by AMS radiocarbon analysis [7].

To examine how different biogenic methane sources have individual isotopic compositions, we obtained two additional biogenic samples from different biogas production facilities within Finland. The results are shown in Table 1. Significant differences are observed between samples for *δ*D, whilst differences in *δ*^13^C, through smaller in magnitude, are also apparent. There is also a clear difference between the biogenic methane samples and the fossil methane sample.

## 4. Discussion

The CH_4_ stable isotope analyser developed for the simultaneous determination of the main, ^13^CH_4_, and CH_3_D isotopologues was tested by determining the biofraction of biogenic and fossil methane mixtures. The biofraction determination provided by the laser spectrometer agreed within 1.5% with the calculated values based on volumetric mixing. A longer measurement time for determining the biofraction was necessary when using the *δ*^13^C values, since the range of delta values between the bio and fossil samples were significantly smaller than for *δ*D.

The uncertainty in the biofraction determination can come from different sources. The optical pathlength utilised in this work was not optimal for either the ^13^C or D transitions monitored, but was rather a compromise to allow the simultaneous measurement of both the *δ*^13^C and *δ*D values at highly elevated methane concentrations. As a result, the large differences in their absorption intensities can be susceptible to system nonlinearities, which are most probably dominated by the combined effects of the detector response and the inadequacy of the Voigt lines shape. In addition to these factors, the calibration strategy employed in this work can limit instrument performance. For environmental applications, where the range of isotopic ratio values is much smaller than the variations measured in this work, one or two-point calibration can be sufficient [32]. For the large range of delta values covered in this study, additional calibration gases would allow for better instrument characterisation, and therefore more accurate determination of the biofraction. In spite of these effects, the instrument performance is comparable to that reported by AMS using the ^14^C method.

One requirement for achieving the level of uncertainty we report here is prior knowledge of the isotopic composition of the source material. However, it is still possible to estimate the biogenic content in mixed bio-fossil mixtures using stable isotopes without this data. The *δ*^13^C and *δ*D values in Table 1 display greater depletion in heavier isotopologues in the biogenic methane relative to fossil methane, which is a finding consistent with other studies [11,12,13,14] and indicative of clear variation between the isotopic signatures of biogenic methane and fossil methane. However, it is important to note that using any approximation of the isotopic signature of the starting materials would introduce significant additional uncertainty into the biofraction value.

The requirement of knowledge of the isotopic composition of the starting material is not unique to the stable isotope approach. To determine biofraction to a similar precision using the ^14^C method, the radiocarbon content of the biogenic fraction of a bio-fossil sample should be determined. Due to temporal and spatial variations in atmospheric ^14^C values (particularly since the 1950s), determining this reference value is not always trivial [7]. However, the uncertainty introduced through approximation of the ^14^C reference value is smaller than the uncertainty introduced through approximation of the source isotopic composition in the stable isotopologues method.

In terms of suitability to stable isotope measurements in industrial applications, the fast analysis, small footprint, and low gas consumption of this instrument makes optical spectroscopy a valid alternative to mass spectrometry for biogas and natural gas applications. Whilst the potential for biogenic content determination is demonstrated here, this instrument would be highly suitable for the online process monitoring of laboratory or industrial scale biogas production processes. Future improvements in instrument stability, calibration, and data-acquisition technology are expected to improve the analysis precision closer to the values reported by commercial optical isotope analysers (currently below 0.1‰ for *δ*^13^C and 0.5‰ for *δ*D values), and further decrease the analysis time.

## Figures and Tables

**Table 1:** Results of isotopic analysis of three biogenic methane samples and one fossil.

Sample	Bio 1 *	Bio 2	Bio 3	Fossil *
*δ*^13^C [‰]	−57.7	−54	−65	−45.6
*δ*D [‰]	−367.3	−370	−324	−203.3

* Analysed with isotope ratio mass-spectrometry (IRMS) and used as reference gases.
